# Management of long segment anterior urethral stricture (≥ 8cm) using buccal mucosal (BM) graft and penile skin (PS) flap: outcome and predictors of failure

**DOI:** 10.1590/S1677-5538.IBJU.2017.0083

**Published:** 2018

**Authors:** Gamal A. Alsagheer, Atef Fathi, Mohamed Sayed Abdel-Kader, Ahmed M. Hasan, Omar Mohamed, Osama Mahmoud, Ahmad Abolyosr

**Affiliations:** 1 Department of Urology, Qena Faculty of medicine, South Valley University, Egypt

**Keywords:** Urethral Stricture, Oral Mucosal Absorption, Penis

## Abstract

**Purpose:**

To evaluate the surgical outcome and predictors of failure of substitution urethroplasty using either dorsal onlay buccal mucosal (BM) graft or ventral onlay penile skin flap (PS) for anterior urethral stricture ≥ 8cm.

**Patients and methods:**

Between March 2010 and January 2016, 50 patients with anterior urethral stricture ≥ 8 cm were treated at our hospital. The surgical outcome and success rate were assessed. The predictors of failure were analyzed using multivariate analysis. Failure was considered when subsequent urethrotomy or urethroplasty were needed.

**Results:**

Dorsal onlay BM graft was carried out in 24 patients, while PS urethroplasty in 26 patients. There was no significant difference between both groups regarding patients demographics, stricture characteristics or follow-up period. One case in the BM group was lost during follow-up. Stricture recurrence was detected in 7 (30.4%) patients out of BM group while in 6 (23.1%) patients out of PS group (p value= 0.5). No significant differences between both groups regarding overall early and late complications were observed. Occurrence of early complications and the stricture length were the only predictors of failure in univariate analysis, while in multivariate analysis the occurrence of early complications was only significant.

**Conclusion:**

On short-term follow-up, both dorsal onlay BM graft and ventral onlay PS flap urethroplasty have similar success rates. However, BM graft has a potential advantage to reduce operative time and is also technically easier. The surgeon should avoid early local complications as they represent a higher risk for failure.

## INTRODUCTION

The gold standard treatment option for anterior urethral stricture not amenable by anastomotic urethroplasty is tissue transfer using either flap or graft (buccal, intestinal mucosa and tissue engineered). The problem is that no single technique is suitable to all conditions and most should be well known by the urologist dealing with this disease (1–4).

Substitution urethroplasty is usually done as a one stage procedure using flap or graft as an onlay patch after stricturotomy, while a 2-stage procedure may be required in cases with severely narrowed lumen or with inadequate blood supply or tissue coverage (5).

Although many studies have discussed the substitution urethroplasty for short segment anterior urethral stricture, only limited studies investigated the management of long segment stricture which is a challenging problem (6). The definition of long segment is not standardized yet in the literature; some studies used 8 cm or 9 cm as a cut off (6, 7), while others defined it as more than one stricture site (8).

Nowadays, BM graft is the most commonly used one from all available grafts due to its easily harvesting with less morbidity to the donor site (9). It has a success rate that reached up to 82.5% for long segment stricture. There is no difference in the success rates between ventral or dorsal placement in the bulbar urethra, but it is not appropriate to use ventral onlay in penile urethra or in long segment stricture (10).

Ventrally placed circular penile fascio-cutaneous flap described by McAninch in 1998 (11) has been advocated for management of stricture segment up to 15 cm without affecting the function or cosmoses with a high success rate and it should be used as an onlay rather than tabularized flap (12).

To our knowledge, few studies compared penile skin flap versus buccal mucosa graft as an onlay substitution for reconstruction of long segment anterior urethral stricture and also the predictors of failure are still not defined in the literature.

## PATIENTS AND METHODS

This is a retrospective study of 56 patients who underwent onlay substitution urethroplasty for long segment anterior urethral stricture ≥ 8 cm between March 2010 and January 2016. Those with unhealthy penile skin, balanitis xerotica obliterans stricture, extremely narrow or obliterated urethra or with history of previous urethroplasty were excluded and only 50 patients were included in our study. The institutional review board approved the study and all patients signed an informed consent. Patient's demographics including age and body mass index (BMI), detailed medical and surgical histories were collected. Preoperative Qmax and stricture characteristics using retrograde urethrogram were recorded for all patients.

### Intervention

Patients underwent either BM urethroplasty or ventral onlay PS flap urethroplasty; selection was based on surgeon preference and experience as we have two teams, each specialized in performing one procedure.

**Figure 1:**
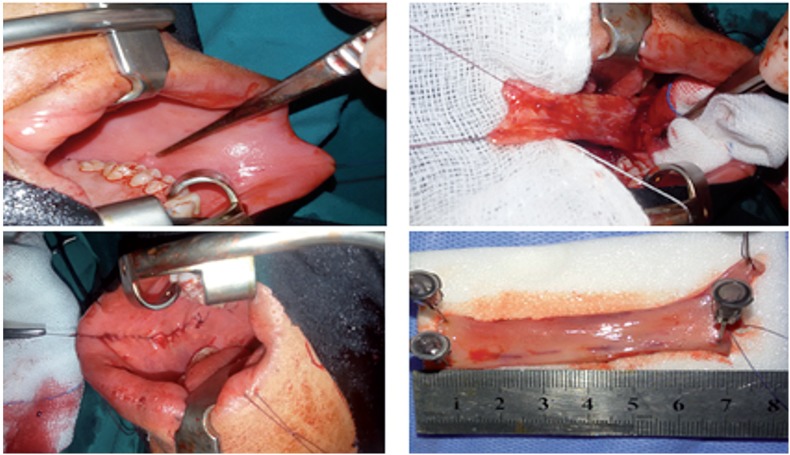
Harvesting of inner cheek buccal mucosa graft.

**Figure 2:**
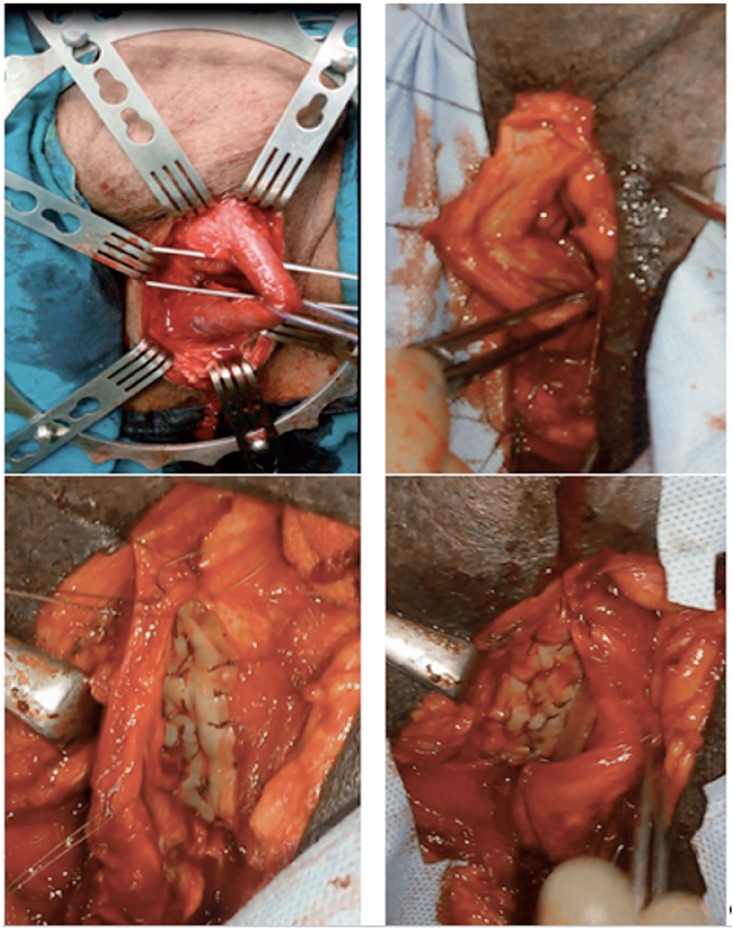
Dorsal onlay buccal mucosa graft augmentation.

BM urethroplasty was carried out as described by Barbagli et al. (13). BM grafts were obtained either from the inner cheek, lower lip or both according to the length of the stricture (Figure-1). The procedure was carried out while the patient was in lithotomy or supine positions according to the site of stricture under general anesthesia with nasal intubation to allow buccal graft harvesting. The urethra was dissected from the corpora cavernosa, then the dorsal surface of the urethra was approached by rotation of the urethra 180 degrees. The urethra was opened until healthy urethral mucosa to assess the length of the stricture. The graft was defatted, fenestrated, and sutured over the corpora cavernosa using 4-0 vicryl stitches in continuous manner over 16 Fr silicone catheter (Figure-2). The procedures were carried out by 2 teams; one for harvesting of buccal graft and another team for urethroplasty.

**Figure 3:**
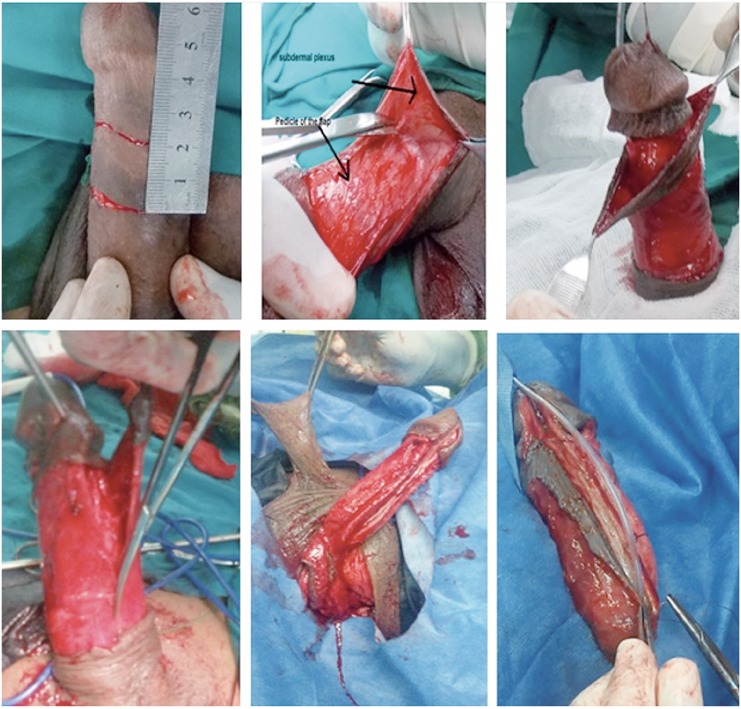
Ventral onlay circular penile flap augmentation.

Ventral onlay PS flap urethroplasty was carried out as described by McAninch et al. (11). Stricturotomy was done ventrally until healthy urethral tissue. According to the length of the urethral stricture, appropriate length circular penile fascio-cutaneous flap was prepared. A sub-coronal circumferential incision was used to harvest the distal penile circular flap with a width ranged from 15 to 20 mm according the available urethral plate. Initially, the dissection was started between the subdermal skin and the dartos fascia then a plane between the dartos fascia and the superficial lamina of Buck's fascia was created up to the base of the penis. The flap as well as the vascular pedicle were then incised in longitudinal fashion to provide a long skin strip. New urethra was created over 16 F urethral silicon catheter with 4/0 Vicryl suture water tight closure without tension and the anastomosis was covered by surrounding fascia (Figure-3). We were careful to put the penis in stretched position to avoid redundancy.

In both groups; suction drain was left at the perineal incision only for 2 days. Silicon catheter 16-F and supra pubic catheter were left in place for 3 weeks. Broad spectrum parental antibiotic was used one hour before the procedure and was continued for 5 days postoperatively. Then, an oral antibiotic was continued until removal of the catheter.

### Follow-Up

Urethral catheter was removed on 21th postoperative day, while the suprapubic catheter was left in place for a few days to ensure a satisfactory voiding before removal. Early postoperative complications were recorded for all patients.

All patients were followed-up at 3, 6, 9, and 12 months postoperatively. At each visit, patients were asked about any urinary symptoms with assessment of residual urine by ultrasound and the late complications were recorded. Retrograde urethrogram was carried out after catheter removal then after 3 months or on demand. Uroflowmetry was carried out at 6 and 12 months. Flexible cystoscopy was carried out for all patients with a suspicious of stricture recurrence. Stricture recurrence that required subsequent urethrotomy, periodic dilatation or urethroplasty was considered failure.

Differences between the groups were assessed using Student t-test for continuous variables, and Chi square test for categorical variables. Logistic regression analysis was used to determine independent predictors of failure after urethroplasty. P value ≤ 0.05 indicates significance. All statistical analyses were performed using SPSS software version 16 (Chicago, IL, USA).

## RESULTS

Among 50 patients, 31 presented difficulty and 13 patients presented irritative lower urinary tract symptoms (LUTS) while 6 cases presented chronic retention. The mean (SD) age, BMI and stricture length were 45 (9.5) years, 27.8 (5.2) Kg/m^2^ and 11.2 (3.6) cm for all patients respectively. The most common site of stricture was bulbo-penile in 68% followed by penile urethra in 22% and bulbar in only 10%. The most common cause of stricture was inflammatory then traumatic and idiopathic causes. Regarding medical co-morbidities, only 2 patients were diabetic (one in each group), 3 patients were hypertensive (2 in BM group and one in PS group), while no patients had cardiac or peripheral vascular diseases. Therefore, we excluded medical co-morbidity from the univariate analysis.

Dorsal onlay BM graft urethroplasty was carried out in 24 patients, while PS urethroplasty in 26 patients. There was no significant difference between both groups regarding patient's demographics, number of patients who underwent previous VIU, number of urehtrotomies, stricture characteristics and preoperative Qmax (Table-1).

**Table 1:** Pre-operative patient's characteristics.

		BM urethroplasty (24 patients)	PS urethroplasty (26 patients)	P value
Age (years), Mean (SD)		44.3 (8.5)	45.2 (10.5)	0.7
BMI (Kg/m^2^), Mean (SD)		27.5 (4.5)	28 (5)	0.8
Intraoperative stricture length (cm), Mean(SD)		11.9 (3.8)	10.7 (2.6)	0.2
Preoperative Qmax (mL/sec)		6.5 (3.4)	7 (2.4)	0.2
Previous VIU, Patients No. (%)		11 (45.8)	10 (38.5)	0.5
Number of previous urethrotomies, Mean (SD)		1.6 (0.8)	1.4 (0.75)	0.4
**Stricture site, Patients No. (%)**				
	Bulbar	2 (8.3%)	3 (11.5)	0.9
	Penile	5 (20.8%)	6 (23)	
	Bulbo-penile	17 (70.8)	17 (65.3)	
**Etiology, Patients No. (%)**				
	Idiopathic	5 (20.8)	6 (23)	0.7
	Traumatic	8 (33.3)	10 (38.5)	
	Inflammatory	11 (45.8)	10(38.5)	

In Table-2 illustrate the operative outcome. The mean ± SD operative time was higher in PS than BM urethroplasty (240.3 ± 45.6 vs. 199.7 ± 51.2, P value = 0.00). The mean (SD) follow-up period was 17.4 (6.6) and 15.7 (7) months for BM group and PS group, respectively (p value = 0.3) with only one case in the BM group lost during follow-up. Recurrence of stricture was detected in 7 (30.4%) patients of BM group while in 6 (23.1%) patients of PS group; 8 patients were managed with redo-urethroplasty while VIU was sufficient in 5 cases. The mean Qmax (SD) at 1 year of follow-up was 15.1 (1.5) and 14.5 (2.7) in BM and PS groups, respectively (p value =0.3). No significant differences for early complications that include hematoma and infection between both groups were observed. Penile skin necrosis occurred in 5 (19%) cases in PS group, which increased the overall early complications in PS group up to 38.4%. Both groups were comparable in relation to late complications. Fistula occurred in 2 (7.6%) patients from PS group and in only 1 (4.3%) patient from BM group (p value = 0.6). Post-micturation dribbling occurred in 6 (23%) cases in PS group, while in 3 (13%) cases only in BM group (p value = 0.3). The univariate analysis for predictors of failure in all 49 patients showed that only lengthy strictures (p = 0.02) and occurrence of early complications (p = 0.00) were predictors of failure (Table-3). For multivariate analysis, only occurrence of early complications is the independent predictor of stricture recurrence (Table-4).

**Table 2:** Surgical outcome.

	BM urethroplasty (24 patients)	PS urethroplasty (26 patients)	P value
Operative time (min.), Mean (SD)	199.7 (51.2)	240.3 (45.6)	0.00
Follow-up duration (mon.), Mean (SD)	17.4 (6.6)	15.7 (7)	0.3
Qmax, (mL/sec), 6 months, Mean (SD)	16 (1.9)	15.4 (2.8)	0.9
Qmax, (mL/sec), 1 year, Mean (SD)	15.1 (1.5)	14.5 (2.7)	0.3
Stricture recurrence, Patients No. (%)	7 (30.4%)	6 (23.1%)	0.5
**Early Complication, Patients No. (%)**	7 (30.5%)	5 (19.2%)	0.4
Hematoma	3 (13%)	2 (7.7%)	
Wound infection	4 (17.5%)	3 (11.5%)	
**Late complications, Patients No. (%)**	4 (17.4)	7 (27)	0.3
Fistula	1 (4.3)	2 (7.6)	
Post void dribbling	3 (13)	6 (23)	
Pseudo-diverticulum	0	2 (7.6)	

**Table 3:** Univariate analysis for predictors of failure.

		Success (36 patients)	Failure (13 patients)	P value
Age (years), Mean (SD)		44.2 (10.2)	47 (6.9)	0.3
Stricture length(cm), Mean(SD)		9.3 (2.5)	12.6 (4.4)	0.02
Preoperative Qmax (mL/sec), Mean (SD)		7.1 (2.9)	6.2 (2.3)	0.1
**Stricture site, Patients No. (%)**				0.07
	Bulbar	5 (13.9)	0	
	Penile	10 (27.8)	1 (7.7)	
	Bulbo-penile	21 (58.3)	12 (92.3)	
**Etiology, Patients No. (%)**				0.15
Idiopathic		7 (19.4)	3 (23.1)	
Traumatic		16 (44.4)	2 (15.4)	
Inflammatory		13 (36.1)	8 (61.5)	
Previous VIU, Patients No. (%)		12 (33.3 %)	8 (61.5)	0.07
Number of previous urethrotomies, Mean (SD)		1.3 (0.7)	1.8 (0.8)	0.08
Early complications, Patients No. (%)		6 (16.7%)	10 (76.9)	0.00

**Table 4:** Multivariate analysis for predictors of failure.

		OR (95% CI)	P value
Stricture length		1.26 (0.92-1.73)	0.2
**Early complications**			
	No	Referent	0.007
	Yes	5.1 (1.8-9.1)	

## DISCUSSION

The repair of long segment urethral stricture is a difficult procedure. Many techniques and materials have been advocated; however, there is still no agreement concerning the best choice (14).

The main cause of stricture in our study was inflammatory (42%), followed by iatrogenic (36%), then idiopathic (22%), while in the developed countries trauma represents the most common cause (15).

In our study, the success rate was comparable between both groups and this copes well with the literature; all previous studies that compared both techniques for a short segment to a panurethral stricture didn't find any difference (6, 16). The published success rate of substitution urethroplasty using either flap or graft is around 85 % (16, 17), while it's slightly lower for the long segment or panurethral stricture. Yue-Min Xu et al. reported an overall success rate of 76.9% for 65 patients with stricture segment > 8 cm at a mean follow-up of 4.8 years using different techniques (18). Kulkarni et al. treated 117 patients with 14 cm mean stricture length using dorsal onlay BM graft and they reported 83.7% success rate at a median follow-up of 59 months (8). On the other hand, McAninch and Morey reported 79% success rate in 66 patients by using circular penile skin flap, the mean stricture length was 9.08 cm with a mean follow-up of 41 months (11). The initial success rate in our study is slightly lower than the previous reports; however, the overall success rate after additional VIU in 5 patients was satisfactory.

Mean operative time (SD) was significantly shorter in BM group than PS group: 199.7 (51.2) and 240.3 (45.6) minutes, respectively (p value = 0.0) and this is in accordance to literature (19). The shorter operative time in BM group is related to presence of two teams who worked simultaneously and also PS is a technically demanding procedure that requires more time for flap dissection.

In our study, an overall 34.6 % of patients developed early post-operative complications, while 22.5% developed late complications and this incidence is similar to previous studies (19, 20). The overall early and late complications were higher in PS group versus BM group; however, the difference was not significant probably due to the small sample size. Previous studies showed significant higher complications for the flap versus the graft; Al-Qudah et al. reported 37 % incidence of early and late complications following BM urethroplasty and 60 % following PS urethroplasty (20). Warner et al. stated that the complication rate is higher in the fascio-cutaneous cohort compared with those without a flap (32% versus 14%, resp.; *P* = 0.02) (6).

Penile skin necrosis occurred in 5 (19%) cases of PS group while for sure not in BM group. The necrosis was mild and healed spontaneously within 1 month in 3 cases, while 2 cases developed infection and stricture recurrence later on. The incidence of necrosis varies in the literature from 4 % to 27.2% (11, 16), it usually is a mild self-limited condition that results from alteration of the skin blood supply from the dissection of the vascular dartos.

In BM group, only 3 patients developed post-micturition drippling, while for sure the incidence was higher for PS group as 6 patients developed drippling, 2 of them had an associated pseudo-diverticulum but none of them had bothered symptoms that required intervention. The low incidence of pseudo-diverticulum is related to that we always use a skin flap width from 1.5 to 2 cm with putting it under stretch to avoid redundancy. We reported 3 cases of urethral-cutaneous fistula in our study (2 cases in PS group and only one case in BM group). They were successfully closed at six months postoperatively with satisfactory results. Fistula was avoided by multiples layers closure over the suture line and using of bipolar diathermy to avoid tissue ischemia. No penile chordee, torsion or shortness were reported in our patients due to the wide and more proximal dissection of the flap till become freely mobile.

We analyzed the predictors of failure among both groups; in the univariate analysis only the length of stricture (p = 0.02) and occurrence of early complications (p = 0.00) were predictors of failure. For multivariate analysis only occurrence of early complications is the independent predictor of stricture recurrence.

Although the stricture length was associated with recurrence in the multivariate analysis in most of the previous studies, it was significant only in our study in the univariate analysis but this may be related to the wider range of stricture length they used (21, 22). In a multivariate analysis conducted by Warner et al. who included only patients with long segment stricture similar to our study, the length was not significant (6). Although the etiology of stricture is not correlated with the recurrence in our study, previous studies showed a positive correlation (15, 24).

Occurrence of early complications in our study was the only independent factor for recurrence in the multivariate analysis, to our knowledge it's the first study to show that. Alterations of flap or graft blood supply by hematoma and infection are important risk factors for failure and these should be taken seriously. Preoperative antibiotic according to culture and adequate postoperative antibiotic are mandatory. Patients with severe difficulty with high residual urine before surgery may require suprapubic catheter for a time. Adequate intraoperative hemostasis using bipolar electrocautery, adequate tamponade postoperative and measures that reduce postoperative erection are also mandatory.

We realize that this study has several limitations, first being retrospective in nature, small sample size that prevented us from subgroup analysis, absence of objective methods to detect the extent of spongiofibrosis which can affect the surgical outcome of urethroplasty and finally the short period of follow-up.

## CONCLUSIONS

On short-term follow-up, both ventral onlay PS urethroplasty and dorsal onlay BM urethroplasty have similar success rates for repair of long anterior urethral stricture; however, PS urethroplasty is associated with more operative time and morbidity. Occurrence of early complications is an important risk of failure and should be avoided as much as possible.

### Ethical approval

All procedures performed in studies involving human participants were in accordance with the ethical standards of the institutional and/or national research committee and with the 1964 Helsinki Declaration and its later amendments or comparable ethical standards.

### Informed consent

Informed consents including the procedure and possible complications were taken from all parents.
